# Anlotinib combined with etoposide for platinum-resistant recurrent ovarian cancer: A case report

**DOI:** 10.1097/MD.0000000000020053

**Published:** 2020-05-15

**Authors:** Li Sun, Meng Yang, Xuan Zhang, Hua Li, Lingying Wu, Yuzi Zhang, Shangli Cai

**Affiliations:** a Departments of Gynecological Oncology, National Cancer Center/National Clinical Research Center for Cancer/Cancer Hospital, Chinese Academy of Medical Sciences and Peking Union Medical College, Beijing; b Departments of Gynecological Oncology, National Cancer Center/National Clinical Research Center for Cancer/Cancer Hospital l & Shenzhen Hospital, Chinese Academy of Medical Sciences and Peking Union Medical College, Shenzhen; c The Medical Department, 3D Medicines Inc, Shanghai, People's Republic of China.

**Keywords:** angiogenesis, anlotinib, etoposide, ovarian cancer, platinum-resistant

## Abstract

**Introduction:**

Platinum-resistant ovarian cancer is characterized by its poor prognosis and limited treatment options. Angiogenesis plays a fundamental role in the development of drug-resistance in ovarian cancer. Anlotinib, a novel oral multi-targeted tyrosine kinase inhibitor which targets a board spectrum of angiogenesis-associated growth factor receptors, has shown promising anti-tumor efficacy in clinical trials. Herein, we report a case of ovarian cancer treated with anlotinib plus etoposide after secondary cytoreductive surgery.

**Patient concerns:**

A 45-year-old female with primary platinum-resistant ovarian cancer who progressed rapidly after the first cytoreductive surgery, the second cytoreductive surgery, and several lines of treatment. The patient refused to receive intravenous chemotherapy any more.

**Diagnosis:**

Primary platinum-resistant ovarian cancer.

**Interventions:**

The oral combination treatment of anlotinib (12 mg, qd) and etoposide (100 mg, qd) were delivered.

**Outcomes:**

Finally, the patient was responsive to the orally treatment of anlotinib combined with etoposide. The patient has been alive with no evidence of disease progression for 18 weeks.

**Conclusion:**

Our case suggests that oral treatment of anlotinib combined with etoposide which is acceptable and convenient, may be an additional option for the management of platinum-resistant ovarian cancer.

## 1 Introduction

Ovarian cancer is the leading cause of mortality in malignant gynecological tumors.^[1]^ Ovarian cancer is considered as one of the platinum-sensitive tumors and platinum containing chemotherapy are standard treatment of ovarian cancer. Although the majority of patients with ovarian cancer are responsive initially to the standard first-line platinum-based chemotherapy, 20% to 30% of patients never achieve a response in the primary setting who are considered as primary platinum-resistant.^[2]^ Effective treatment strategies are limited for patients who progressed or had persistent disease on primary platinum-based chemotherapy. For platinum-resistant ovarian cancer, non-platinum-based agents are recommended, such as docetaxel, etoposide, liposomal doxorubicin, and paclitaxel. However, the abnormal tumor vasculature may hinder the delivery of therapeutic agents into the inside of large tumor masses. Therefore, removal of large chemo-resistant tumors could exert favorable impact on the following treatment of recurrent disease. Although it is generally accepted that secondary cytoreductive surgery can be considered for patients with recurrent platinum-sensitive disease, retrospective studies also indicated that secondary cytoreductive surgery could provide clinical benefit in patients with platinum-resistant disease with a longer post-relapse survival.^[3,4]^

Anti-angiogenesis is also considered as promising target for the treatment of platinum-resistant ovarian cancer.^[5]^ Bevacizumab, a FDA-approved monoclonal antibody targeting vascular endothelial growth factor (VEGF), and other compounds targeting tyrosine kinases are two major types of anti-angiogenesis agents. Anlotinib (AL3818) is a novel oral multi-targeted tyrosine kinase inhibitor on tumor angiogenesis and growth.^[6]^ Anlotinib has a broad spectrum of inhibitor effects on targets such as vascular endothelial growth factor receptors 2/3 (VEGFR2/3), fibroblast growth factor receptor 1-4 (FGFR1-4), platelet-derived growth factor receptors α/β (PDGFR α/β), c-kit, and Ret.^[7]^ The phase II/III randomized, double-blind ALTER-0303 trial has demonstrated that patients treated with anlotinib exhibited significantly longer overall survival compared with patients treated with placebo (9.6 vs 6.3 months; hazard ratio 0.68; *P* = .0018).^[8]^ Anlotinib has been approved in China as a single drug therapy for patients with locally advanced or metastatic non-small cell lung cancer (NSCLC) who have progressed after the second line therapy or beyond.^[6]^ In addition, several clinical trials are ongoing to evaluate the efficacy of anlotinib in a variety of malignancies such as gynecologic tumors, colorectal cancer, and gastric cancer.^[6]^ NCT02558348 is an ongoing phase I/II clinical trial assessing anti-tumor efficacy of anlotinib single treatment in patients with ovarian cancer, endometrial cancer, or cervical cancer. Another phase I/II clinical trial, NCT02584478, is designed to evaluate the efficacy of anlotinib plus standard platinum-based chemotherapy in recurrent or metastatic endometrial, ovarian, fallopian, primary peritoneal, or cervical carcinoma. However, there is no report of the efficacy of anlotinib in ovarian cancer. Herein, we present a case of a female patient with primary platinum-resistant ovarian cancer treated with anlotinib combined with etoposide after secondary cytoreductive surgery in our hospital.

## 2 Case report

**Figure 1:**
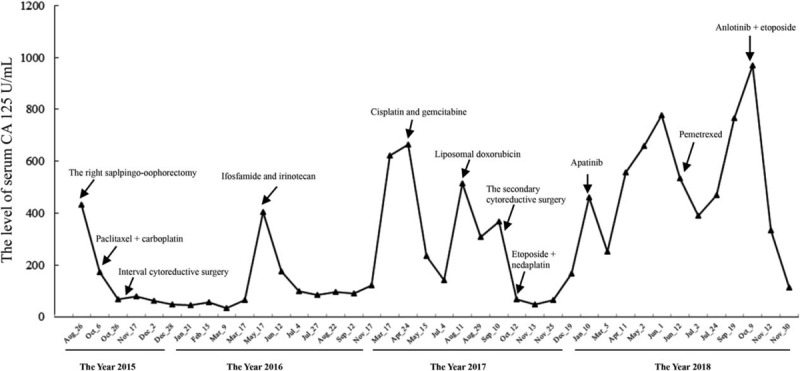
The levels of serum CA 125 of the patient.

A 45-year-old female was diagnosed with ovarian serous papillary cystadenocarcinoma after right salpingo-oophorectomy in August 2015. Informed consent was obtained from the patient. After neoadjuvant chemotherapy with two cycles of paclitaxel and carboplatin, she had undergone an interval cytoreductive surgery consisting of left salpingo-oophorectomy, omentectomy, and pelvic lymphadenectomy. She was considered as optimal debulked stage IIIc disease according to the findings during the surgery. Following this, she received seven cycles of adjuvant chemotherapy with paclitaxel and carboplatin. Seven months after the initial treatment, she experienced the first relapse with the elevated serum CA125 level (Fig. 1) and the positron emission tomography/computed tomography (PET/CT) images of multiple metastasis in the internal lymph node of left breast, mediastinum, peritoneum, and pelvic cavity. Then, she was recommended to take ifosfamide and irinotecan. After seven cycles, she refused to receive chemotherapy. One year after the first relapse, she experienced the second relapse diagnosed by PET/CT images of multiple new metastases sites including vagina, rectum, inguinal region, para-aorta, and peritoneum. After three cycles of cisplatin and gemcitabine, a third relapse appeared rapidly with CT scan images of new mass in bladder, lower ureter, sigmoid colon, and pararectal space.

The patient was referred to our institution (Shenzhen Hospital) in August 2017. After the discussion by our multi-disciplinary team (MDT), chemotherapy with liposomal doxorubicin was recommended and a secondary cytoreductive surgery should be considered. Molecular testing was also recommended by the MDT, but no pathogenic alterations of *BRCA1* or *BRCA2* gene was found. After two cycles of liposomal doxorubicin, the patient had undergone the second cytoreductive surgery including appendectomy, resection of pelvic mass, partial rectectomy, pelvic adhesiolysis, distal rectal closure, and proximal sigmoid ostomy. The secondary cytoreductive surgery was also considered optimal. After three cycles of chemotherapy with etoposide and nedaplatin postoperatively, the patient refused intravenous chemotherapy. Then, she was treated with apatinib orally as maintenance treatment for 5 months. The patient provided informed consent for administering apatinib. Six months after initial treatment with liposomal doxorubicin, she experienced the fourth relapse with metastasis in the medial segment (S4) of liver diagnosed by CT scan and she had undergone trascantheter arterial chemoembolization of the hepatic lesion. The treatment of apatinib was discontinued due to the side effects such as hand-foot skin reaction, oral ulcer, and pain. Then, intravenous chemotherapy with pemetrexed was delivered. After three cycles, she refused to receive intravenous chemotherapy any more. Then, the patient progressed rapidly with the markedly elevated CA125 and CT image of new metastasis in left supraclavicular lymph nodes.

**Figure 2:**
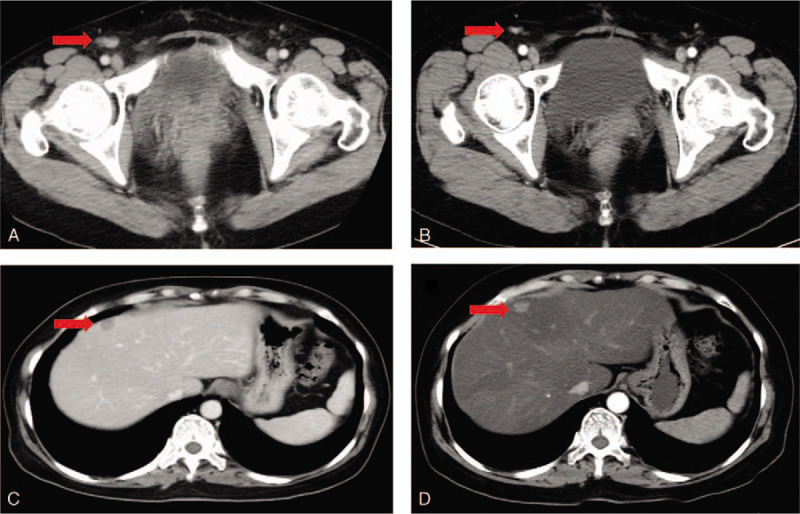
CT image showing metastases before and during the treatment of anlotinib plus etoposide. CT image showing (A) lymph nodes metastases in the right inguinal region (C) liver metastases before treatment of anlotinib plus etoposide (October 9, 2018). CT image showing (B) smaller lymph nodes metastases in the right inguinal region (D) similar liver metastases after eleven weeks of anlotinib plus etoposide treatment (December 24, 2018).

Three months after the last treatment of pemetrexed, the patient began to take anlotinib (12 mg, qd) and etoposide (100 mg, qd) orally. Informed consent was provided by the patients for the treatment of anlotinib. Eleven weeks after the orally combined treatment, the CT scan indicated partial response (PR). Although the liver metastases was similar compared with the previous evaluation, the involved lymph nodes in mesenterium and right inguinal region were shrinking compared with the previous evaluation (Fig. 2). The serum CA125 also decreased significantly from 969 to 84.8 U/mL. Now the patient has been alive with no evidence of disease progression for 18 weeks. In addition, the patient experienced grade II hand-foot skin reaction. No major toxic effects appeared. All the treatments were listed in Table 1.

**Table 1:**
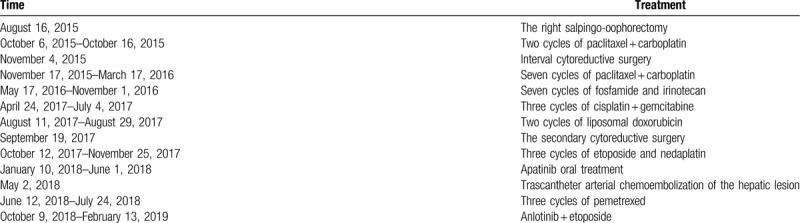
All treatment that the patient has received.

## 3 Discussion

We describe a female with primary platinum-resistant ovarian cancer who was responsive to the orally treatment of anlotinib plus etoposide. The patient has been heavily treated and was considered as highly chemo-resistant. The addition of the novel multi-target tyrosine kinase inhibitor to single chemotherapy regimen was considered as a valuable treatment strategy for platinum-resistant ovarian cancer. In addition, during the treatment of this refractory ovarian cancer, the secondary cytoreductive surgery also provided benefit for this chemo-resistant patient.

Ovarian cancer is characterized by its high mortality and advanced diagnosis.^[1]^ Cytoreductive surgery and platinum-based chemotherapy are standard treatment for patients with ovarian cancer. Although the majority of patients with ovarian cancer are responsive initially to the standard first-line platinum-based chemotherapy, 20% to 30% of patients never achieve a response in the primary setting who are considered as primary platinum-resistant.^[2]^ Effective treatment options for patients who are considered as primary platinum chemo-resistance are limited and the prognosis of patient with primary platinum chemo-resistant ovarian cancer was significantly reduced. That said, major challenge remains for the treatment of primary chemo-resistance patients. Clinical trials, best supportive care, and recurrence therapy are suggested by the NCCN Guidelines version 2.2018 (Epithelial Ovarian Cancer/Fallopian Tube Cancer/Primary Peritoneal Cancer) for such special population and highly individual basis should be taken into consideration.

For platinum-resistant ovarian cancer, non-platinum-based agents are recommended, such as docetaxel, etoposide, liposomal doxorubicin, and paclitaxel. However, the abnormal tumor vasculature may hinder the delivery of therapeutic agents into the inside of large tumor masses. Therefore, removal of large chemo-resistant tumors could exert favorable impact on the following treatment of recurrent disease and the goal of complete cytoreduction has extended to the setting of recurrent ovarian cancer. However, the role of cytoreductive surgery in management of recurrent ovarian cancer remains controversial. Only retrospective studies have demonstrated the survival benefit from secondary cytoductive surgery in recurrent setting.^[3,4]^ A retrospective study have demonstrated a significantly prolonged median time to first progression (12 months vs 3 months, *P* = .016) in platinum-resistant ovarian cancer receiving secondary cytoreductive surgery compared with patients receiving chemotherapy alone. Several case reports also indicated a survival benefit in women with platinum-resistant disease treated with secondary cytoreductive surgery combined with hyperthermic intraperitoneal chemotherapy (HIPEC).^[9,10,11]^ In our case who was heavily treated and progressed rapidly after several lines of chemotherapy, the secondary cytoreductive surgery plus sequential maintenance treatment provided a progression-free survival (PFS) of 5.8 months.

Multiple mechanisms may contribute to platinum resistance in ovarian cancer.^[12,13]^ The microenvironment of tumor including blood vessels, inflammatory cells, stromal cells, and other components plays essential roles in the process of tumor progression and the development of drug resistance.^[13]^ Angiogenesis-associated growth factor receptors are most widely studied and considered as promising treatment targets.^[5,14]^ A retrospective study revealed that higher angiogenesis-associated growth factor receptors such as PDGFR-beta and VEGFR-2 levels were associated with the platinum resistance status of ovarian cancer.^[15]^ Several studies also demonstrated that the increased levels of angiogenesis-associated growth factor receptors were associated with inferior prognosis in ovarian cancer.^[15,16,17]^ FDA has approved bevacizumab, a monoclonal antibody targeting VEGF, for the treatment of platinum-resistant, recurrent ovarian cancer.^[18,19]^ Although bevacizumab could prolong PFS in patients with platinum-resistant disease, no significant benefit of overall survival (OS) was achieved. Multi-target tyrosine kinases compounds have also exhibited promising anti-tumor efficacy in clinical trials. The phase II trial MITO 11 demonstrated a significant improved progression-free survival in the treatment of pazopanib which inhibits VEGFR1-3, PDGFRα/β, and PDGFR 1-3 plus paclitaxel arm (6.35 months) relative to paclitaxel arm (3.49 months) in platinum-resistant, recurrent ovarian cancer (HR 0.42, *P* = .0002).^[20]^ Other clinical trials evaluating platinum-based chemotherapy in combination with multi-target tyrosine kinases inhibitors such as nintedanib and cediranib also achieved therapeutic efficacy in platinum-sensitive ovarian cancer.^[21,22]^

Anlotinib (AL3818) is a novel oral multi-targeted tyrosine kinase inhibitor which could highly selectively target several angiogenesis growth factor receptors.^[6,23]^ Anlotinib has a broad spectrum of inhibitor effects on targets such as vascular endothelial growth factor 2/3 (VEGFR2/3), fibroblast growth factor receptor 1–4 (FGFR1-4), platelet-derived growth factor receptors α/β (PDGFR α/β), c-kit, and Ret.^[7]^ It has been reported that the anti-angiogenesis efficacy of anlotinib is stronger than several anti-angiogenesis regimen, including sunitinib and sorafenib, because anlotinib can target more targets than these drugs.^[7,24]^ The anti-angiogenesis activity of anlotinib has been illustrated in vitro and in vivo.^[7,25,26]^ In addition, anlotinib can highly bound to plasma and has a lower incidence of toxic effects in terms of grade 3 or higher.^[27]^ Anlotinib has shown promising efficacy in the treatment of advanced NSCLC, metastatic renal cell carcinoma, soft tissue sarcoma, and advanced medullary thyroid cancer.^[8,28,29,30]^ Anlotinib has been approved in China as a single drug therapy for patients with locally advanced or metastatic non-small cell lung cancer (NSCLC) who have progressed after the second line therapy or beyond.^[6]^ Thus, anlotinib may contribute to overcome the chemo-resistant status of ovarian cancer. NCT02558348 is an ongoing phase I/II clinical trial assessing anti-tumor efficacy of anlotinib single treatment in patients with ovarian cancer, endometrial cancer, or cervical cancer. Another phase I/II clinical trial, NCT02584478, is designed to evaluate the efficacy of anlotinib plus standard platinum-based chemotherapy in recurrent or metastatic endometrial, ovarian, fallopian, primary peritoneal or cervical carcinoma.

Etoposide, a cost-effective oral agent, has a long history in the treatment of platinum-resistant ovarian cancer. The reported median response duration of etoposide single drug treatment was 4.3 months and the median progression-free interval was 5.7 months in patients with platinum-resistant ovarian cancer.^[31]^ In addition, etoposide was also effective in patients with paclitaxel-resistant ovarian cancer.^[31]^

We used anlotinib in combination with etoposide to control the platinum-resistant refractory ovarian cancer after the secondary cytoreductive surgery. This oral treatment is acceptable, convenient, and cost-effective without the need for hospital admissions or infusion pumps. Hand-foot skin reaction, hypertension, proteinuria, and triglyceride elevation were the most common adverse events in the treatment of anlotinib. In our case, the patient only experienced grade 2 hand-foot skin reaction which was well managed.

## 4 Conclusion

In conclusion, the therapeutic strategy of anlotinib plus etoposide may provide an additional treatment options for patients with platinum-resistant ovarian cancer. Further investigations are warranted to optimize the efficacy of anlotinib plus etoposide in the treatment of patients with platinum-resistance ovarian cancer.

## Acknowledgments

Thank for the patient for agreeing to the publication of the case.

## Author contributions

**Conceptualization:** Li Sun, Shangli Cai

**Data curation:** Meng Yang, Xuan Zhang, Hua Li, Lingying Wu

**Supervision:** Li Sun

**Writing – original draft:** Li Sun, Yuzi Zhang

**Writing – review & editing:** Li Sun, Shangli Cai, Meng Yang, Xuan Zhang, Lingying Wu, Hua Li
